# Usefulness of clinical predictors for preoperative screening of deep vein thrombosis in hip fractures

**DOI:** 10.1186/s12891-017-1582-5

**Published:** 2017-05-22

**Authors:** Kitchai Luksameearunothai, Paphon Sa-ngasoongsong, Noratep Kulachote, Sorawut Thamyongkit, Praman Fuangfa, Pongsthorn Chanplakorn, Patarawan Woratanarat, Chanyut Suphachatwong

**Affiliations:** 1 Department of Orthopedics, Faculty of Medicine Ramathibodi Hospital, Mahidol University, 270, Rama VI Road, Ratchathewi, Bangkok, 10400 Thailand; 2 Department of Radiology, Faculty of Medicine Ramathibodi Hospital, Mahidol University, Bangkok, Thailand; 3 Chakri Naruebodindra Medical Institute, Faculty of Medicine Ramathibodi Hospital, Mahidol University, Bangkok, Thailand; 4 Department of Orthopaedics, Faculty of Medicine Vajira Hospital, Navamindrahiraj University, Bangkok, Thailand

**Keywords:** Preoperative screening, Deep vein thrombosis, Hip fracture, Wells score, Caprini score

## Abstract

**Background:**

Recent studies showed that preoperative deep vein thrombosis (DVT) was common after hip fracture (HF), and preoperative DVT screening has been recommended for preventing the fatal DVT-related complications, especially in elderly HF patients with high surgical risk. However, to our knowledge, no previous studies have demonstrated the correlation between the clinical risk predictors and preoperative DVT. Therefore, this study aimed to correlate those clinical predictors related to DVT risk assessment with the incidence of preoperative DVT.

**Methods:**

A prospective study was conducted, between July 2015 and June 2016, in 92 HF patients. All patients were evaluated for the DVT-related risk, as patients’ characteristics, clinical signs, D-dimer, DVT risk assessment score (Wells score and Caprini score), and then underwent doppler ultrasonography preoperatively. The incidence of preoperative DVT was correlated with each clinical risk predictor, and then significant factors were calculated for diagnostic accuracy.

**Results:**

The average patients’ age was 78 ± 10 years. Sixty-eight patients (74%) were female. The incidence of preoperative DVT was 16.3% (*n* = 15). The median time from injury to doppler ultrasonography was 2 days (range 0–150 days). DVT group showed a significantly higher in Wells score and Caprini score compared to the non-DVT group (*p* < 0.05 all). Sensitivity and specificity of Wells score ≥ 2 and Caprini score ≥12 were 47 and 81, and 93 and 35%, respectively.

**Conclusion:**

DVT risk assessment may be helpful for stratifying the risk of preoperative DVT in elderly HFs. Those with Caprini score ≥ 12 should be screened with doppler ultrasonography preoperatively. Those with Wells score 0–1 had low risk for preoperative DVT, so the surgery could perform without delay.

## Background

Geriatric patients with hip fracture (HF) are at very high risk for the development of venous thromboembolism (VTE) complication, as deep vein thrombosis (DVT) and pulmonary embolism (PE), which is a principal cause of perioperative mortality and morbidity [1, 2]. Recently, there has been a markedly growing attention in diagnosis of preoperative DVT after HF due to the concern of the potentially lethal complications from fresh thrombus emboli such as acute massive intraoperative PE, and sudden cardiac arrest [3–10]. Previous studies have shown that the incidence of preoperative DVT in the HF patients, by using preoperative screening either doppler ultrasonography (USG) or venography with or without computerized tomography (CT), varied from 2.6 to 17.3%, and could be as high as 62% particularly in the HF patients who had delayed operation more than 2–3 days [4–10]. As a result, those HF patients with preoperative DVT were safely treated with either inferior vena cava (IVC) filter placement or anticoagulant medication before undergoing the HF surgery without any VTE-related postoperative complications [5–7, 9, 10]. Moreover, this preoperative screening could be helpful for perioperative management as using perioperative mechanical DVT prophylaxis (such as pneumatic intermittent compressive device) in the HF patients who did not have preoperative DVT [11]. Therefore, due to this noteworthy incidence and its possibly fatal consequence, many previous studies recommended that all HF patients, especially those who had delayed operation, should be routinely investigated for preoperative DVT [4–8].

However, the application of the preoperative DVT screening strategy, by using either contrast venography or doppler USG, in all HF patients may be difficult. Although contrast venography is considered as a gold standard tool for DVT diagnosis, this method still has many drawbacks due to its invasiveness such as pain on injection site, contrast medium idiosyncratic reactions, nephrotoxicity, and contrast-induced DVT [12] that is not suitable for elderly HF patients. Therefore, doppler USG has become a popular and acceptable tool for DVT diagnosis due to its non-invasiveness nature. However, the difficulties of routine preoperative DVT screening with doppler USG are the requirement of experienced radiologist on 24-h basis, additional cost of investigation, and the risk of delayed HF surgery resulting in prolonged hospital stay, and significantly higher mortality and morbidity [13, 14]. Besides, some HF patients with low risk for DVT may not need preoperative screening with doppler USG and could be treated with early hip fracture surgery as soon as possible. Nonetheless, to our knowledge, the application of the DVT risk assessment stratification in preoperative DVT diagnosis has still been not established. Therefore, the aim of this study was to evaluate the usefulness of the standard DVT risk assessment method for predicting the preoperative DVT in the patients with hip fracture.

## Methods

### Study population

This was a single-centered prospective observational study in an academic university hospital, from July 2015 to June 2016, in the patients with hip fracture. The study protocol was approved by our institutional review board (Protocol number ID 12-58-43). The inclusion criteria were the patients who presented with hip fractures and had admitted for definitive treatment in our hospital. The exclusion criteria were severe dementia or uncooperative for preoperative assessment protocol, multiple fractures, pathologic fracture from metastasis, and peri-implant or periprosthetic fracture patients.

### Study protocol and data collection

After admission and allocation into the study, the patients would be asked for complete history and physical examination information. Basic patients’ characteristic and hip-fracture related data, such as age, gender, body mass index (BMI), pre-existing comorbid disease, history of active smoking, side of injury, fracture type, time of injury and mechanism of injury were recorded. The type of fracture was collected as intracapsular and extracapsular fracture. Mechanism of injury was recorded as low-energy, such as simple fall on the ground, and high-energy, such as motorcycle accident. Age and comorbid diseases were calculated into Charlson comorbid index (CCI) [15] Physical examinations related to hip fracture and diagnosis of DVT, such as swollen leg and pitting leg edema, were collected. Then all patients were evaluated with DVT risk assessment score, as well as Wells score [16] and Caprini score [17], by one of the authors who was an experienced trauma surgeon (K.L.). Preoperative laboratory tests including D-dimer were conducted at the time of admission. The preoperative doppler USG was scheduled and performed by one of the authors (P.F.), who was an experienced musculoskeletal radiologist, within 24 h after admission, and then the time from injury to doppler USG was calculated in day. The diagnosis of DVT solely depended on the result of doppler USG, and was classified into acute and chronic DVT. The criteria for acute DVT were homogeneous, smooth hypo-echoic signal with deformable under compression, and dilated vein distal to thrombus. The criteria for chronic DVT were heterogeneous, irregular, synechiae echogenic signal with non-deformable vein, normal or small vein size, thickened venous wall and recanalization with the presence of the collateral vessels.

###  Statistical analysis

Statistical analysis was performed using Statistical Package of Social Sciences (SPSS) software version 18.0. Normally distributed continuous data were presented as mean and standard deviation. Student *t*-test was used for variables with equal variance, and Welch test was used for variables with unequal variance. Non-normally distributed continuous data were presented as median and interquartile range, and compared with Mann–Whitney *U* test. Categorical data were presented as proportion and compared with Fisher’s exact test or Chi-square test as appropriate. Significant difference was considered if *p*-value < 0.05. The significant factors for prediction of DVT diagnosis were presented as relative risk (R.R.) and their 95% confidence interval (C.I.). Sensitivity, specificity, positive predictive value and negative predictive value were calculated for the significant diagnostic test for DVT.

## Results

**Fig. 1:**
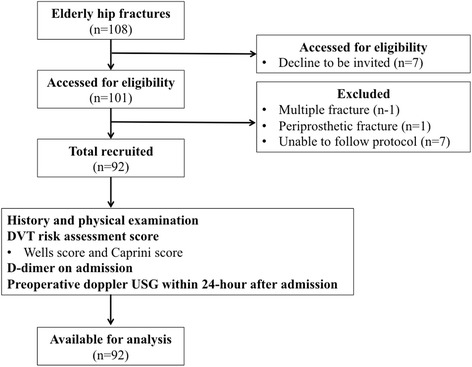
flow diagram of this study

A total of 92 hip fracture patients were enrolled into this study as shown in Fig. 1. Among these patients, 68 of them (74%) were female and the average patients’ age was 78 years (range 47–96 years). Forty-five cases (49%) were classified as extracapsular fracture (44 intertrochanteric fractures and 1 subtrochanteric fracture) (Table 1). The incidence of DVT was 16.3% (15 patients), including 10 patients with acute DVT (10.9%) and 5 patients with chronic DVT (5.4%) (Tables 1 and 2). Regarding 10 patients with acute DVT, nine of them had proximal DVT (90%) and the other one had distal DVT (10%). Five patients (50%) with acute DVT had intracapsular hip fracture, whereas the other five patients (50%) had extracapsular hip fracture. All patients with acute DVT were treated with IVC filter or subcutaneous enoxaparin injection before HF surgery. For five patients with chronic DVT, all of them had extracapsular hip fracture (100%) and having proximal DVT that were treated with subcutaneous enoxaparin injection. The mean Wells score and Caprini score were 0.9 (range 0–4) and 12.1 (range 10–15). The median time from injury to doppler USG was 2 days (range from 0 to 150 days), and the median D-dimer was 4343 ng/mL (range 296–47,600 ng/mL).

**Table 1:** Characteristics of the study population and difference between DVT group and non-DVT group

	Total (*n* = 92)	DVT group (*n* = 15)	Non-DVT group (*n* = 77)	*p*-value
Age, year Ω	78 ± 10	81 ± 10	78 ± 9	0.29
Female gender ♦	68 (73.9)	12	56	0.75
BMI, kg/m^2^ c	22.6 ± 4.5	23.5 ± 6..0	22.4 ± 4.1	0.49^w^
Right side ♦	52 (56.5)	8	44	0.78
Extracapsular fracture ♦	45 (48.9)	10	35	0.16
High-energy mechanism ♦	5 (5.4)	1	4	1.00
Active smoker ♦	3 (3.3)	2 (13.3)	1 (1.3)	0.009*
CCI Ω	5.7 ± 2.3	6.5 ± 2.7	5.5 ± 2.2	0.11
Swollen leg ♦	9 (9.8)	5	4	0.005*
Pitting edema ♦	6 (6.5)	3	3	**0.05**
Time to doppler USG, day	2 (1–7)	3 (1.25–6.5)	2 (1–7.25)	0.61^m^
Wells score Ω	0.9 ± 0.9	1.5 ± 1.1	0.8 ± 0.8	0.05*
Caprini score Ω	12.1 ± 1.4	12.8 ± 1.3	11.9 ± 1.3	0.02*
D-dimer, ng/mL ■	4343 (1766–11433)	4530 (2675–8874)	4258 (1611–12080)	0.68^m^

*BMI* body mass index, *CCI* Charlson comorbidity index

Ω; value presented as mean ± standard deviation

♦; value presented as number of cases (percentage)

■; value presented as median (interquartile range)

^w^; *p*-value calculated from Welch test

^m^; *p*-value calculated from Mann-Whitney *U* test

*; significant value with *p* < 0.05

**Table 2:** Difference between acute DVT and chronic DVT group

	Acute DVT (*n* = 10)	Chronic DVT (*n* = 5)	*p*-value
Age, year Ω	79 ± 11	84 ± 9	0.34
Female gender ♦	7	5	0.51
BMI, kg/m^2^ Ω	21.6 ± 4.8	27.4 ± 6.9	0.08
Right side ♦	5	3	1.00
Extracapsular fracture ♦	5	5	0.10
High-energy mechanism ♦	1	0	1.00
Active smoker ♦	2	0	0.52
CCI Ω	6.6 ± 3.1	6.4 ± 2.1	0.90
Swollen leg ♦	4	1	0.60
Pitting edema ♦	2	1	1.00
Time to doppler USG, day Ω	5.8 ± 6.0	3.4 ± 3.7	0.44
Wells score Ω	1.8 ± 1.1	0.8 ± 0.8	0.11
Caprini score Ω	13.0 ± 1.5	12.4 ± 0.5	0.41
D-dimer, mg/mL Ω	5921 ± 5871	8514 ± 6905	0.46

*BMI* body mass index, *CCI* Charlson comorbidity index

Ω; value presented as mean ± standard deviation

♦; value presented as number of cases (percentage)

Regarding the predictive factors for developing preoperative DVT, the DVT group showed a significant difference in history of active smoking, clinical signs (swollen leg, and pitting edema), and DVT risk assessment scores (Wells score, and Caprini score) compared with non-DVT group (*p* < 0.05 all). However, there was non-significant difference in the patients’ characteristics (age, gender, BMI, fracture type, mechanism of injury, and CCI), time from injury to doppler USG, and D-dimer level between both groups (*p* > 0.05 all) (Table 1).

Table 2 demonstrated the differences between acute DVT and chronic DVT group. No significant difference in the patients’ characteristics, clinical signs, time from injury to doppler USG, risk assessment score, and D-dimer level had been found between both groups (*p* > 0.05 all). However, BMI in acute DVT group was lesser, but non-significantly, than those in chronic DVT group (*p* = 0.08).

Table 3 showed the diagnostic accuracy of using the DVT risk assessment score to predict preoperative DVT. The significant correlation between the DVT risk assessment scores and preoperative DVT was found with Wells score ≥ 2 points and ≥ 3 points, and Caprini score ≥ 12 points and ≥ 13 points (*p* < 0.05 all). Sensitivity and specificity from Wells score ≥ 2 points were 47 and 81%, and those from Wells score ≥ 3 points were 13 and 99%, respectively. Sensitivity and specificity from Caprini score ≥ 12 points were 93 and 35%, and those from Caprini score ≥ 13 points were 60 and 73%, respectively.

**Table 3:** Diagnostic accuracy of using Wells score and Caprini score for preoperative DVT

	R.R. (95% C.I.)	*p*-value	Sensitivity	Specificity	PPV	NPV
Wells score						
≥ 1	2.42 (0.63–9.29)	0.20	80.0	37.7	20.0	90.6
≥ 2	3.62 (1.13–11.54)	0.03*	46.7	80.5	31.8	88.6
≥ 3	11.69 (0.99–138.44)	0.05*	13.3	98.7	66.7	85.4
Caprini score						
≥ 11	2.84 (0.34–23.56)	0.33	93.3	16.9	17.9	92.9
≥ 12	7.56 (0.94–60.64)	0.05*	93.3	35.1	21.9	96.4
≥ 13	4.00 (1.27–12.61)	0.02*	60	72.7	30	90.3
≥ 14	2.16 (0.50–9.30)	0.3	20	89.6	27.3	85.2

*R.R.* relative risk, *C.I.* confidence interval, *PPV* positive predictive value, *NPV* negative predictive value

*; significant value with *p* < 0.05

## Discussion

Recent studies have demonstrated that a significant proportion of the patients with hip fracture (HF) already had DVT preoperatively, and stressed out the importance of DVT diagnosis by using preoperative screening with doppler USG or venography [5–7, 9, 10]. However, preoperative DVT screening in all HF patients with a gold standard contrast venography might not be appropriate because of its invasiveness (such as pain, contrast medium reaction, and contrast-induced renal toxicity), and lack of venous access in some elderly HF patients. Moreover, routine preoperative DVT screening requires experienced radiologist on 24-h basis, with additional imaging cost and also having the risk of complications from the surgical delay. Therefore, the preoperative evaluation with the other simpler methods, such as DVT risk assessment score, to stratify the risk of preoperative DVT in the HF patients and use of preoperative screening in these high-risk cases, would be a more appropriate option. However, to our best knowledge, none of previous studies have been shown the correlation of preoperative DVT in the HF patients with the other assessment methods including the clinical signs and the DVT risk assessment score, and the applicability of DVT risk assessment score for diagnosing preoperative DVT. Therefore, this study aimed to evaluate the correlation between the preoperative DVT and these predicting methods, and the usefulness of these methods for predicting the preoperative DVT in the HF patients.

The incidences of overall preoperative DVT, acute DVT, and chronic DVT after HF in this study were 16.3% (15 patients), 10.9% (10 patients), and 5.4% (5 patients), respectively. Regarding to the incidence of acute preoperative DVT, our finding was comparable with the previous studies [6–10]. However, one previous study had demonstrated the incidence was only as 1.4% among the HF patients who were admitted within 72 h after injury, while increased as 13.3% among those who were admitted later than 72 h after injury [5]. These findings implied that the time delay from HF injury to admission and receiving the surgical treatment should be a significant predictive factor for the development of acute preoperative DVT [4, 5], and might be one responsible cause for the variation of the incidence of acute preoperative DVT among the literature. Therefore, to prevent VTE complication, the healthcare system should pay their attention for developing the model of geriatric emergency care, such as rapid response and urgent referral of elderly HF patients, and prompt diagnosis with fast-tracking preoperative medical clearance, for decreasing this unnecessary time delay and the incidence of acute preoperative DVT.

Our study unexpectedly demonstrated that some HF patients (5.4%) had concomitant asymptomatic chronic DVT which had never been diagnosed before sustained the injury, and also had not received the same attention as acute preoperative DVT among the previous literature. However, this incidence of undiagnosed chronic DVT was comparable to previous studies in non-HF elder medical patients [18, 19]. Oger E et al. found that the prevalence of asymptomatic DVT on admission among the elderly medical patients, using compression doppler USG within 48 h after admission, was 5.5%, and particularly increased to 17.8% among those patients aged over 80 years [18]. This implied that the incidence of undiagnosed asymptomatic and chronic DVT in elderly HF patients was not sporadic, and should be significantly correlated with increasing age resulting in a higher risk for developing perioperative VTE complication, as acute on chronic DVT or recurrent DVT [20, 21]. Thus, it is worth to mention the importance of preoperative screening of the undiagnosed chronic DVT in these high surgical risk geriatric patients, especially in those who had no DVT history but having positive signs and symptoms for DVT, or preoperative Caprini score ≥ 12 (Tables 1 and 2). Moreover, we also recommended using DVT chemoprophylaxis, in the elderly HF patients with chronic DVT, before HF surgery in every cases.

Our results also showed that preoperative DVT was significantly associated with history of active smoking, clinical signs and DVT risk assessment score as Wells score and Caprini score, but was not significantly associated with the time after injury to doppler USG, or D-dimer level (Table 1). Regarding to the correlation between history of active smoking and DVT, our finding was comparable with the previous studies that cigarette smoking was a significant risk factor for VTE [22, 23]. The non-significant difference between preoperative DVT after HF and the time after injury to doppler USG might be explained by two reasons; first, the sample size of our study may be too small to detect any difference in this risk factor. Secondly, our study populations might have selection bias because some elderly HF patients had been referred from the other hospitals, to our academic university hospital, due to the multiple comorbidities and high risk for surgery. Moreover, our results showed that the one-thirds of the patients with preoperative DVT in this study were chronic DVT that had not been diagnosed before. However, there was non-significant difference of preoperative risk factors, clinical signs, DVT risk assessment score, or D-dimer level between the patients with acute DVT or chronic DVT (*p* > 0.05 all, Table 2). The non-significant difference between preoperative DVT after HF and D-dimer level could be explained by the normally increase of D-dimer level after fracture [24]. This is because the fracture bleeding and hematoma could result in the significant change in coagulation parameter such as greater in fibrinogen and D-dimer level compared to the control, especially in the larger size bone as femoral fracture [24].

Furthermore, our results demonstrated that preoperative DVT may be predictable by careful assessment of the patients’ clinical signs and risk factors following the standard clinical probability scoring system. Regarding to the diagnostic accuracy of these DVT risk assessment scores, Wells score should be considered as the test for excluding preoperative DVT due to its low sensitivity and high specificity, and Caprini score should be used as the screening test due to its high sensitivity and low specificity. Moreover, we found that using Wells score ≥ 2 points, and Caprini score ≥ 12 points would be the appropriately significant cut-off levels as shown in Table 3. Our findings showed that if the patients with hip fracture had Caprini score ≥ 12 points, the relative risk for having preoperative DVT was 7.56 times significant higher than those with Caprini score < 12 points and we suggested using doppler USG to diagnose preoperative DVT before operation. Also if the patients with hip fracture had Wells score only 0–1 point, the preoperative screening with doppler USG may not be needed due to the high negative predictive value of 89% (Table 3). With this risk stratification strategy, the patients with low risk for preoperative DVT could be safely and effectively treated with urgent hip fracture surgery without any delay from the unnecessary investigation. Based on our data, this strategy would reduce the need of the preoperative DVT screening by half, and therefore significantly decrease the radiologists’ excessive workload. Although the application of this risk stratification strategy might not change the requirement of 24-h radiologist service in large trauma center or university hospital, this application should be helpful for reducing the workload in the hospital with high-volume HF surgery or proving the better preoperative management in smaller hospital which usually lack of available 24-h experienced radiologist.

Our study also had some limitations. Firstly, we used preoperative doppler USG screening instead of gold standard contrast venography. This could be considered as not the “best available gold standard”, and might affect the true incidence of preoperative DVT in this study. However, this contrast venography has its own limitations and the inherent risks of serious complications which were not feasible for the elderly patients who had high risk for complications, which therefore, rendered its use in general clinical settings. Moreover, previous studies showed that doppler USG had an acceptable diagnostic accuracy particularly in the symptomatic proximal DVT [25, 26], and was suitable for preoperative DVT screening [5–7, 9]. Secondly, all preoperative doppler USG in this study was performed by only one experienced musculoskeletal radiologist (P.F.), without any double blind control, which might affect the accuracy of doppler USG. Thirdly, our sample size might be too small. Regarding to our findings on the prevalence of preoperative DVT (16.3%), the required sample size, in which the marginal error not exceed than 3 with 95% confidence interval, would be 575 patients. Fourthly, due to the nature of university hospital, some of our elder HF patients were not the newly diagnosed cases, but were the referral cases from the other hospitals due to high risk for surgery and multiple comorbidities requiring advanced medical care or financial problem. Therefore, the incidence of preoperative DVT in our study should be slightly higher than the true incidence in the general population by the effect of time delay from HF injury to admission. Further multi-centered prospective studies with larger sample size are required. Lastly, we did not perform serial doppler USG on the postoperative period; so the true incidence of overall perioperative DVT did not show in this study. However, the information about the postoperative DVT and the need of prophylaxis in hip fracture surgery were already established [27].

## Conclusion

Our findings indicate that DVT risk assessment score, as Wells score and Caprini score, could be helpful for stratifying the risk of preoperative DVT in the elderly HFs. We suggest that if the Caprini score is 12 points or more, doppler USG should be performed. Also if Wells score is 0 or 1 points, the risk of preoperative DVT is very low and hip fracture surgery without delay from waiting for other investigations for diagnosing DVT should be considered.

## Abbreviations

BMI: Body mass index
C.I.: Confidence interval
CCI: Charlson comorbid index
CT: Computerized tomography
DVT: Deep vein thrombosis
HF: Hip fracture
PE: Pulmonary embolism
R.R.: Relative risk
USG: Ultrasonography
VTE: Venous thromboembolism
